# Propofol anesthesia decreases the incidence of new-onset postoperative atrial fibrillation compared to desflurane in patients undergoing video-assisted thoracoscopic surgery: A retrospective single-center study

**DOI:** 10.1371/journal.pone.0285120

**Published:** 2023-05-02

**Authors:** Karin Tajima, Kentaro Yamakawa, Yuki Kuwabara, Chika Miyazaki, Hiroshi Sunaga, Shoichi Uezono

**Affiliations:** Department of Anesthesiology, The Jikei University School of Medicine, Tokyo, Japan; Ataturk University Faculty of Medicine, TURKEY

## Abstract

**Background:**

Postoperative atrial fibrillation (POAF) increases postoperative morbidity, mortality, and length of hospital stay. Propofol is reported to modulate atrial electrophysiology and the cardiac autonomic nervous system. Therefore, we retrospectively examined whether propofol suppresses POAF in patients undergoing video-assisted thoracoscopic surgery (VATS) compared to desflurane.

**Methods:**

We retrospectively recruited adult patients who underwent VATS during the period from January 2011 to May 2018 in an academic university hospital. Between continuous propofol and desflurane administration during anesthetic maintenance, we investigated the incidence of new-onset POAF (within 48 hours after surgery) before and after propensity score matching.

**Results:**

Of the 482 patients, 344 received propofol, and 138 received desflurane during anesthetic maintenance. The incidence of POAF in the propofol group was less than that in the desflurane group (4 [1.2%] vs. 8 patients [5.8%], odds ratio [OR]; 0.161, 95% confidence interval (CI), 0.040–0.653, *p* = 0.011) in the present study population. After adjustment for propensity score matching (n = 254, n = 127 each group), the incidence of POAF was still less in propofol group than desflurane group (1 [0.8%] vs. 8 patients [6.3%], OR; 0.068, 95% CI: 0.007–0.626, *p* = 0.018).

**Conclusions:**

These retrospective data suggest propofol anesthesia significantly inhibits POAF compared to desflurane anesthesia in patients undergoing VATS. Further prospective studies are needed to elucidate the mechanism of propofol on the inhibition of POAF.

## Introduction

Atrial fibrillation (AF) is the most common type of arrhythmia after surgery and is associated with increased morbidity, mortality, length of hospital stay, and medical costs [1–5]. Lung surgery is a high-risk surgery for postoperative atrial fibrillation (POAF) in the range of 3% to 20% [1, 2, 4, 6, 7], compared to general surgery (less than 10%) [8, 9]. As risk factors for POAF in lung surgery, increasing age, male sex, and increasing extent of pulmonary resection have been reported [2, 4, 10, 11]. High-risk electrocardiographic findings for POAF, which reflect atrial structural and/or electrical remodeling [12, 13], were also studied in lung and cardiac surgery.

From the anesthetic standpoint, either an intravenous or a volatile anesthetic agent is administered for anesthetic maintenance. The selection of anesthetic agents is generally based on the anesthesiologists’ discretion and/or hospital practices. In lung surgery, clinical advantages for postoperative pulmonary complications between anesthetic agents are comparable [14–16]. Regarding perioperative arrhythmias, several studies showed propofol suppressed supraventricular tachycardia, and propofol modulated atrial electrophysiology, atrioventricular conduction, and cardiac autonomic nervous system [17–19]. This evidence suggests that propofol can potentially contribute to preventing POAF. On the other hand, volatile anesthetic agents have not shown more antiarrhythmic effects than propofol [20–23].

Therefore, we retrospectively examined the effects of propofol on POAF compared to the volatile anesthetic agent with desflurane in video-assisted thoracoscopic surgery (VATS), along with possible underlying mechanisms by some literature reviews.

## Materials and methods

### Ethical approval

The Jikei University Review Board approved this retrospective clinical study, which provided a waiver of patient consent (reception number 30–369 [9390]).

### Patients

From January 2011 to May 2018, we analyzed data for patients with lung disease who underwent VATS at our institution. We excluded patients < 20 years of age, with insufficient preoperative electrocardiography (ECG), or with a history of chronic or paroxysmal AF or congenital heart disease. We also excluded if they underwent converted open thoracotomy or pneumonectomy, received a mixture of propofol and desflurane during anesthesia maintenance, or received general anesthesia alone without epidural anesthesia. The data analyzed were incidence of POAF, age, sex, height and body weight, comorbidities, American Society of Anesthesiologists Physical Status (ASA PS), and preoperative ECG. Comorbidities included hypertension (HT), diabetes mellitus (DM), chronic obstructive pulmonary disease (COPD), and current smoker status (had smoked within the past year). We also assessed electrolyte status on postoperative day (POD) 1.

### Data collection and definitions

We reviewed medical records, anesthesia records, and the intensive care unit (ICU) database to obtain patient background, anesthetic management, surgical procedure, and POAF outcome data. The primary outcome was POAF incidence, defined as new onset within 48 hours after surgery. In addition, we use the Clavien-Dindo classification of surgical classifications (grade I-V) to categorize POAF; grade Ⅰ did not need intervention, grade Ⅱ required pharmacological treatment, grade Ⅲ required surgical interventions, grade Ⅳ was a life-threatening complication, and grade Ⅴ was death [16].

### Preoperative electrocardiography

Preoperative ECG indicates atrial structural and/or electrical remodeling and predicts POAF after lung and cardiac surgery [12, 13]. Therefore, we analyzed preoperative ECG for all patients performed by medical technologists within 6 weeks before surgery to evaluate basic structural substrate. Standard 12-lead ECG was documented with an automatic electrocardiographic recorder (model FX-7432; Fukuda Denshi, Tokyo, Japan) at a paper speed of 25 mm/s. The following parameters were measured: P-wave duration, P-wave amplitude, and QRS duration. The P-wave duration was measured between P-wave onset and offset. The P-wave dispersion was defined as the difference between the longest and the shortest P-wave durations across 12 leads. The longest PR interval was defined as the longest interval in any of the 12 leads measured between the beginning of the P-wave and Q-wave onset. The QRS duration was measured between the Q-wave onset and the J-point, and the longest QRS duration was examined for all leads. In addition, left atrial enlargement (LAE) was defined as a P-wave duration >120 ms at lead II or a terminal negative P-wave in V1 >40 ms wide and >1 mm deep (P-wave terminal force > 40 ms × mm) [24]. LVH was present if the sum of the S-wave in V1 and the R-wave in V5 or V6 >35 mm (Sokolow-Lyon index) [25].

### Perioperative management

Routine monitoring, including ECG, pulse oximetry, noninvasive blood pressure monitoring with continuous arterial pressure via a radial artery, and end-tidal carbon dioxide pressure, was performed from induction to the end of anesthesia. Before anesthetic induction, we routinely inserted an epidural catheter without contraindications for better perioperative analgesia. For anesthetic induction in the propofol group, we administered propofol using a target-controlled infusion device (Terufusion syringe pump system; Terumo, Tokyo, Japan). Fentanyl (1–2 μg/kg) (fentanyl citrate; Sankyo, Tokyo, Japan) and/or remifentanil (0.1–0.25 μg/kg/min) (Ultiva; Janssen Pharmaceutical, Tokyo, Japan) and rocuronium (0.6–1.0 mg/kg) (Eslax; MSD, Tokyo, Japan) were also administered.

In the desflurane group, we administered propofol (1–2 mg/kg) just for induction, and opioids and neuromuscular blockade were administered as described above, followed by inhaled desflurane (0.6–1.0 minimal alveolar concentration (MAC)) (Suprane; Baxter, Deerfield, IL, US) for maintenance.

Depth of anesthesia was maintained at 40 to 60 by bispectral index (BIS-XP system; Medotronic, Minneapolis, MN, USA) throughout the operation in both groups. Dexamethasone (0.1–0.15 mg/kg, max 6.6 mg) was administered intraoperatively to prevent postoperative nausea and vomiting [26]. The selection of an anesthetic agent and the use of dexamethasone were at the anesthesiologist’s discretion. The type of operation was segmentectomy or lobectomy with lymphoidectomy by VATS.

After surgery, we administered 1–4 mg/kg of sugammadex as needed to antagonize residual neuromuscular paralysis by monitoring with train-of-4 monitoring. After extubation, we transferred all patients to the ICU for overnight observation. Postoperative management, including laboratory data in the ICU, was performed by ICU specialists. Electrocardiographic monitoring was continued 48 hours after surgery, even if the patient returned to the ward the following day.

### Statistical analysis

Quantitative data are presented as median (interquartile range) and qualitative data as number (%). For quantitative data, an unpaired *t* test or Mann-Whitney U test was performed per statistical data normality, and qualitative data were analyzed by chi-square test (if the chi-square test was not eligible for the condition, the Fisher exact probability method was used). After univariate analysis, multivariate logistic regression analysis was examined to investigate the association with POAF.

Given the inherent differences between the two groups, propensity score matching was performed to compare the incidence of POAF. The participants were 1:1 matched with nearest neighbor method without replacement, within caliper <0.1: the propofol group and the desflurane group. The propensity score was calculated by a multivariate logistic regression model involving the following covariates. Covariates included anesthetic agents, age, gender, height, body weight, past history with HT, DM, COPD, current smoker, ASA PS, procedure type, anesthesia time, operation time, one lung ventilation time, bleeding, in-out fluid balance [1, 2, 4, 6, 10, 27], serum potassium [28], and ECG findings of the P-wave dispersion [29], the maximum QRS duration, left atrial enlargement [12], and left ventricular hypertrophy [30]. A *p* value <0.05 was considered statistically significant, and confidence intervals (CIs) were 95%. For all statistical analyses, data were analyzed by EZR version 1.55 (Saitama Medical Center, Jichi Medical University, Saitama, Japan), which is a graphical user interface for R (The R Foundation for Statistical Computing, version 2.7–1, Vienna, Austria) [31].

## Results

### Patients and perioperative management

**Fig 1:**
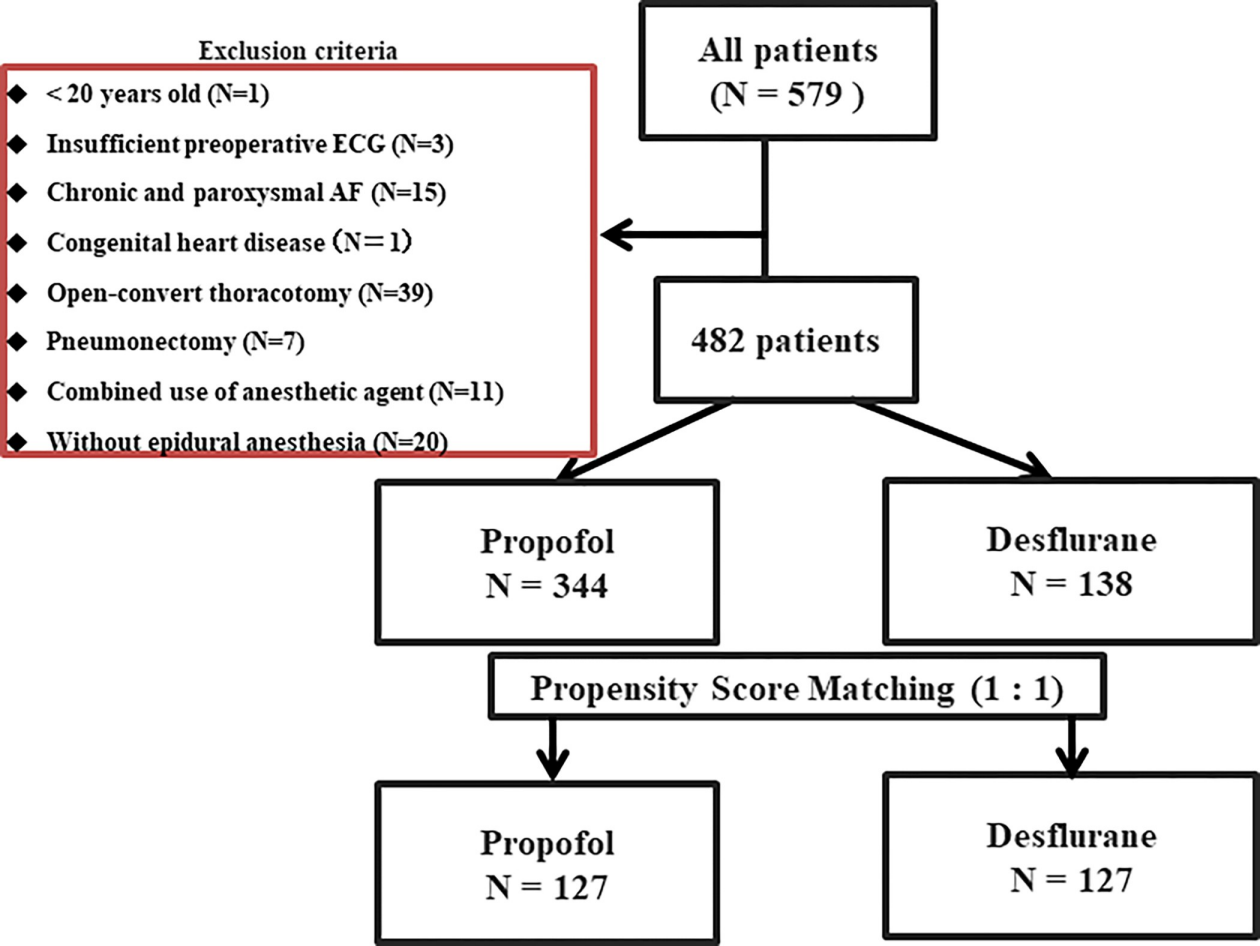
Flow chart of the study population. AF: atrial fibrillation, ECG: electrocardiogram.

A total of 579 patients were identified, and 97 were excluded (Fig 1). Among the remaining 482 patients, 344 received continuous propofol, and 138 were maintained with desflurane for general anesthesia. In the current study, factors that affected the incidence of POAF were age, beta-blocker, and anesthetic agent (Table 1). Patient characteristics, anesthetic management, electrolyte findings, and procedure type between propofol and desflurane groups were summarized in Table 2. Patient characteristics were comparable between groups. However, during the intraoperative period, the propofol group showed significantly longer anesthesia, longer operation time, longer one-lung ventilation time, and more bleeding than the desflurane group. In addition, electrolyte serum potassium on POD 1 was significantly lower in the propofol group.

**Table 1:** Patient characteristics, anesthetic management, and surgical procedure based on POAF incidence.

		POAF (n = 12)	Non-POAF (n = 470)	*p* value
**Patient characteristics**	**Age (yr)**	75 (68–76)	67 (61–74)	0.013
	**Gender male, N (%)**	10 (83.3)	288 (61.3)	0.852
	**Height (cm)**	168 (162–172)	163 (156–169)	0.956
	**Body weight (kg)**	69 (63–75)	59 (52–67)	0.169
	**HT, N (%)**	6 (50.0)	203 (43.2)	0.244
	**DM, N (%)**	2 (16.7)	73 (15.5)	0.393
	**COPD, N (%)**	2 (16.7)	57 (12.1)	0.686
	**Current smoker, N (%)**	2 (16.7)	35 (7.4)	0.147
	**ASA PS Ⅰ, N (%)**	1 (8.3)	78 (16.6)	0.627
	**Ⅱ, N (%)**	11 (91.7)	372 (79.1)	
	**Ⅲ, N (%)**	0 (0)	20 (4.3)	
	**Beta-blocker, N (%)**	3 (25.0)	20 (4.3)	0.012
**Type of surgery**	**Lobectomy, N (%)**	11 (91.7)	414 (88.1)	0.897
	**Segmentectomy, N (%)**	1 (8.3)	56 (11.9)	
**Anesthetic management**	**Propofol, N (%)**	4 (30.8)	340 (72.3)	0.044
	**Desflurane, N (%)**	8 (69.2)	130 (27.7)	
	**Anesthesia time (min)**	351 (280–417)	349 (301–409)	0.559
	**Operation time (min)**	260 (212–331)	264 (213–320)	0.496
	**OLV time (min)**	229 (201–323)	249 (201–304)	0.835
	**Bleeding (ml)**	85 (50–138)	50 (0–100)	0.296
	**In-out fluid balance (ml/kg/hr)**	2.45 (1.89–3.16)	3.58 (2.41–5.05)	0.072
**Electrolyte on POD1**	**Serum potassium (mEq/L)**	4.20 (4.00–4.43)	4.00 (3.80–4.20)	0.091
**ECG markers**	**The P-wave dispersion (ms)**	54 (32–75)	62 (30–74)	0.436
	**The longest PR duration (ms)**	176 (159–209)	176 (160–190)	0.953
	**The longest QRS duration (ms)**	111 (105–115)	108 (102–116)	0.997
	**Left atrial enlargement (%)**	5 (41.7)	143 (30.4)	0.943
	**Left ventricular hypertrophy (%)**	0 (0)	72 (15.3)	0.993

Data are presented as median (interquartile range) or number (percent).

POAF: postoperative atrial fibrillation, HT: hypertension, DM: diabetes mellitus, COPD: chronic obstructive pulmonary disease, ASA PS: American society of anesthesiologist physical status, OLV: one-lung ventilation, ECG: electrocardiogram POD: postoperative day, PSM: propensity score matching, SMD: standardized mean difference

**Table 2:** Patient characteristics, anesthetic management, and surgical procedure (Before and after propensity score matching).

		Before PSM				After PSM			
		Propofol (n = 344)	Desflurane (n = 138)	*p* value	SMD	Propofol (n = 127)	Desflurane (n = 127)	*p* value	SMD
**Patient characteristics**	**Age (yr)**	67 (61–73)	69 (63–75)	0.064	0.175	71 (64–76)	68 (62–75)	0.213	0.161
	**Gender male, N (%)**	217 (63.1)	81 (58.7)	0.407	0.09	75 (59.1)	75 (59.1)	1.000	<0.001
	**Height (cm)**	163 (157–169)	163 (156–169)	0.531	0.064	161 (155–167)	162 (156–169)	0.322	0.121
	**Body weight (kg)**	60 (52–68)	59 (52–66)	0.457	0.077	58 (51–66)	59 (52–66)	0.94	0.003
	**HT, N (%)**	144 (41.9)	65 (47.1)	0.310	0.106	59 (46.5)	55 (43.3)	0.705	0.063
	**DM, N (%)**	46 (13.4)	29 (21.0)	0.051	0.204	29 (22.8)	21 (16.5)	0.269	0.159
	**COPD, N (%)**	41 (11.9)	18 (13.0)	0.759	0.034	16 (12.6)	15 (11.8)	1.000	0.024
	**Current smoker, N (%)**	23 (6.7)	14 (10.1)	0.255	0.125	14 (11.0)	12 (9.4)	0.836	0.052
	**ASA PS Ⅰ, N (%)**	61 (17.7)	18 (13.0)	0.059	0.228	12 (9.4)	18 (14.2)	0.465	0.155
	**Ⅱ, N (%)**	273 (79.4)	110 (79.7)			109 (85.8)	102 (80.3)		
	**Ⅲ, N (%)**	10 (2.9)	10 (7.2)			6 (4.7)	7 (5.5)		
	**Beta-blocker, N (%)**	18 (5.2)	5 (3.6)	0.637	0.078	9 (7.1)	4 (3.1)	0.254	0.179
**Type of surgery**	**Lobectomy, N (%)**	301 (87.5)	124 (89.9)	0.535	0.074	112 (88.2)	113 (89.0)	1.000	0.025
	**Segmentectomy, N (%)**	43 (12.5)	14 (10.1)			15 (11.8)	14 (11.0)		
**Anesthetic management**	**Anesthesia time (min)**	356 (308–423)	331 (280–367)	<0.001	0.411	330 (290–387)	331 (280–370)	0.490	0.056
	**Operation time (min)**	271 (223–331)	242 (197–292)	<0.001	0.374	246 (202–297)	242 (200–293)	0.525	0.035
	**OLV time (min)**	253 (206–315)	227 (191–273)	<0.001	0.337	236 (193–276)	228 (198–277)	0.593	0.059
	**Bleeding (ml)**	50 (0–120)	45 (0–100)	0.001	0.304	50 (0–100)	40 (0–100)	0.438	0.114
	**In-out fluid balance(ml/kg/hr)**	3.65 (2.53–5.08)	3.23 (2.15–4.76)	0.093	0.153	3.54 (2.35–4.94)	3.45 (2.17–4.79)	0.718	0.019
**Electrolyte on POD1**	**Serum potassium (mEq/L)**	4.00 (3.80–4.10)	4.10 (3.90–4.30)	<0.001	0.355	4.10 (3.85–4.20)	4.10 (3.90–4.20)	0.588	0.016
**ECG markers**	**The P-wave dispersion (ms)**	62 (28–75)	63 (34–74)	0.553	0.076	66 (31–78)	62 (34–72)	0.254	0.115
	**The longest PR duration (ms)**	176 (158–190)	178 (160–192)	0.312	0.11	178 (162–192)	176 (160–192)	0.608	0.050
	**The longest QRS duration (ms)**	108 (102–116)	108 (102–116)	0.818	0.028	108 (103–116)	108 (102–117)	0.950	0.073
	**Left atrial enlargement (%)**	101 (29.4)	47 (34.1)	0.327	0.101	41 (32.3)	41 (32.3)	1.000	<0.001
	**Left ventricular hypertrophy (%)**	58 (16.9)	14 (10.1)	0.067	0.197	14 (11.0)	14 (11.0)	1.000	<0.001

Data are presented as median (interquartile range) or number (percent).

HT: hypertension, DM: diabetes mellitus, COPD: chronic obstructive pulmonary disease, ASA PS: American society of anesthesiologist physical status, OLV: one-lung ventilation, POD: postoperative day, ECG: electrocardiogram, PSM: propensity score matching

SMD: standardized mean difference.

After adjustment for propensity score matching, 127 pairs were matched. There was no significant difference between propofol and desflurane groups, and standardized mean differences were less than 0.2 (Table 2 and S1 Data). The intraoperative maximal effect-site target concentration (Ce) of propofol was 2.7 (2.5–3.0) μg/mL, the minimal Ce was 2.5 (2.0–2.6) μg/mL.

### Incidence of POAF

The overall incidence of POAF before propensity score matching (new onset, within 48 hours after VATS) was 12 of 482 patients (2.5%) in this study; 4 (1.2%) in the propofol group, and 8 (5.8%) in the desflurane group. There was less POAF incidence in the propofol group than in the desflurane group (odds ratio [OR]; 0.161, 95% confidence interval (CI), 0.040–0.653, *p* = 0.011) in the present study population. The POAF incidence after propensity score matching was 9 of 254 patients (3.5%) in this study; 1 (0.8%) in the propofol group and 8 (6.3%) in the desflurane group. There was also less POAF incidence in the propofol group than in the desflurane group (OR; 0.068, 95% CI: 0.007–0.626, *p* = 0.018) (Table 3).

**Table 3:** The incidence of postoperative atrial fibrillation.

**Before PSM**	**All**	**Propofol**	**Desflurane**	***p* value**	**SMD**
POAF incidence, N (%)	12/482 (2.5%)	4/344 (1.2%)	8/138 (5.8%)	0.006	0.255
Odds ratio (95% Confidence Interval); 0.161 (0.040–0.653), *p* = 0.011					
**After PSM**	**All**	**Propofol**	**Desflurane**	***p* value**	**SMD**
POAF incidence, N (%)	9/254 (3.5%)	1/127 (0.8%)	8/127 (6.3%)	0.036	0.302
Odds ratio (95% Confidence Interval); 0.068 (0.007–0.626), *p* = 0.018					

POAF: postoperative atrial fibrillation, PSM: propensity score matching

SMD: standardized mean difference

#### Clinical course, classification of surgical complications, and management of POAF

After adjustment for propensity score matching, a total of 6 patients with POAF (67%) recovered to regular sinus rhythm without pharmacologic therapy (grade І) (Table 4). We performed pharmacologic therapy for rate or rhythm control in 3 patients (grade Ⅱ). In the desflurane group, a 78-year-old POAF patient developed transient cardiac arrest due to pulmonary thromboembolism in the general ward. However, the POAF was not supposed to be the direct cause of the incident. Except for the case, patients with POAF returned to the general ward on POD1 with ECG monitoring and experienced no adverse events.

**Table 4:** Clinical data of POAF patients and classification of surgical complications.

Anesthetic agent	Age (yr)	ASA PS	Procedure	Onset	Classification of complication	Treatment	Included after PSM
Propofol	62	2	Lobectomy	POD2	Ⅰ	None	**No**
	83	2	Lobectomy	POD1	Ⅰ	None	**No**
	67	1	Lobectomy	POD1	Ⅱ	Rate and rhythm control	**No**
	75	2	Lobectomy	POD1	Ⅱ	Rate and rhythm control	**Yes**
Desflurane	67	2	Lobectomy	POD1	Ⅰ	None	**Yes**
	68	2	Lobectomy	POD1	Ⅰ	None	**Yes**
	75	2	Lobectomy	POD1	Ⅰ	None	**Yes**
	75	2	Lobectomy	POD1	Ⅰ	None	**Yes**
	75	2	Segmentectomy	POD1	Ⅰ	None	**Yes**
	78	2	Lobectomy	POD1	Ⅰ	None	**Yes**
	69	2	Lobectomy	POD1	Ⅱ	Rate control, Bolus crystalloid	**Yes**
	83	2	Lobectomy	POD1	Ⅱ	Rate control, Magnesium	**Yes**

ASA PS: American society of anesthesiologist physical status, PSM: propensity score matching, POD: postoperative day

## Discussion

There has been little clinical research on POAF incidence and anesthetic agents in lung surgery. Therefore, we investigated the novel comparison between standard anesthetic agents (propofol vs. desflurane) on the incidence of POAF after VATS. Our results showed that the incidence of new-onset POAF after VATS was significantly less for propofol anesthesia than for desflurane anesthesia.

Non-cardiac thoracic surgery is a high-risk procedure for POAF, with a reported incidence of 3% to 40% [2, 4–6, 32]. For lung surgery, the incidence of POAF is reported to be 10% to 20% for open thoracotomy, whereas it is 3% to 20% for VATS [32–35]. The lower incidence of POAF in VATS is thought to be due to that being a less-invasive procedure [27, 33]. In the present study, before matching, the incidence of POAF in the propofol group (1.2%) was less than the overall incidence (2.5%), and less than previously reported [32–35]. In subgroup analysis for the Perioperative Ischemic Evaluation (POISE) trial, although an anesthetic agent was not described, the incidence of POAF was predicted by 5.3% from the risk model, which includes age and type of surgery [5]. It is consistent with the desflurane group by 5.8% in our study before and similar even after matching (6.3%). Therefore, we speculate that this study’s difference in POAF incidence was due to propofol’s beneficial effect, not desflurane’s adverse effect.

In lung surgery, as standard practice, an intravenous or volatile anesthetic agent is administered for anesthetic maintenance. The outcome of major postoperative complications, including pulmonary complications, has been comparable between anesthetic agents [14–16]. Among volatile anesthetics, desflurane is mainly used in our hospital because of its short-acting work and is tolerated to be administered in a clinical setting [36, 37]. In addition, some studies have claimed that a high concentration of desflurane has been linked to tachycardia in healthy volunteers due to increased sympathetic stimulation [38, 39]. Nonetheless, as pointed out in the review [40], the difference between volatile anesthetics was believed to be limited during balanced maintenance of anesthesia with less than 1MAC of desflurane in our study. No difference was also observed between sevoflurane and desflurane anesthesia regarding postoperative pulmonary complications in lung surgery [28].

### Two steps to POAF

**Fig 2:**
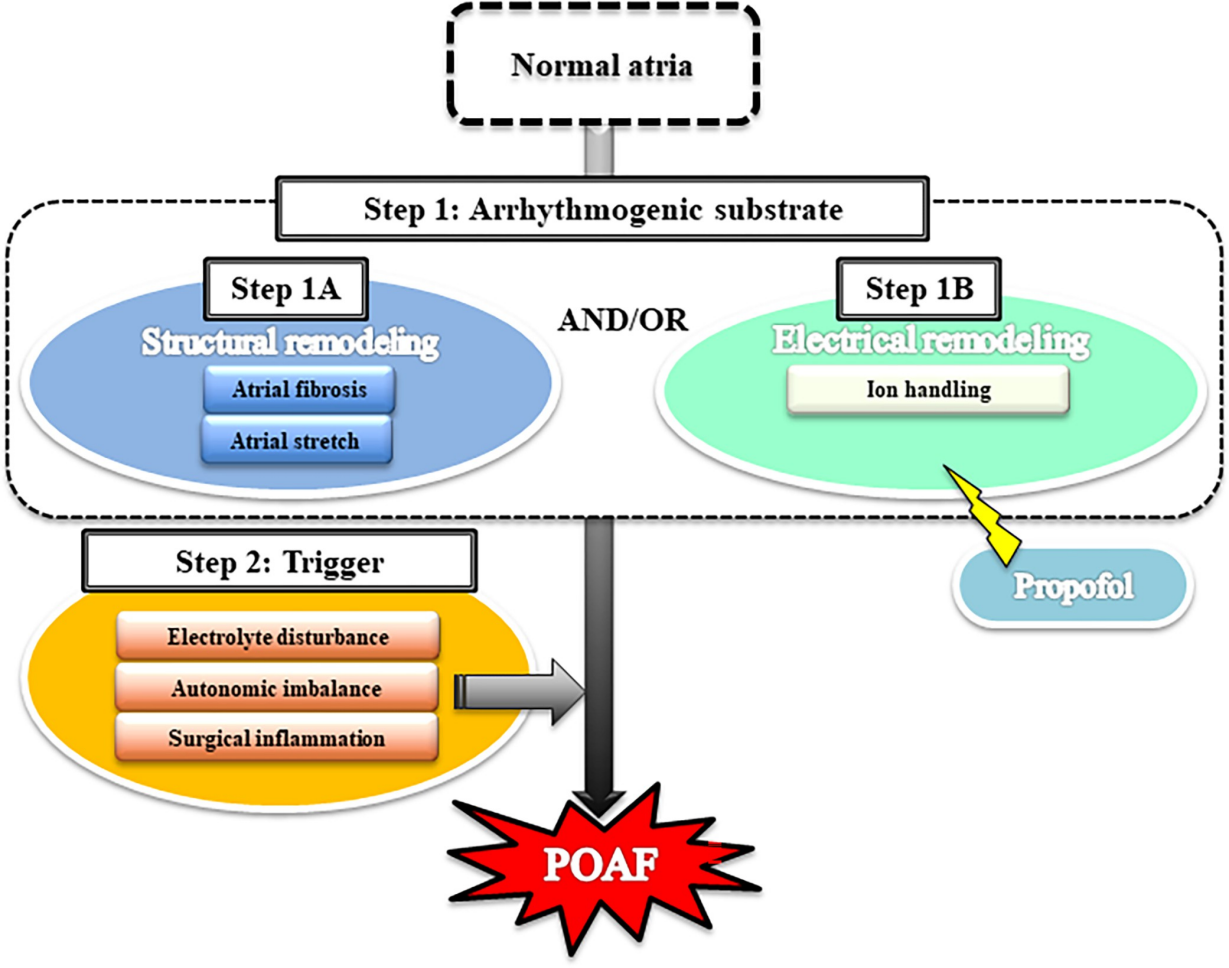
Schematic of the mechanism underlying the development of postoperative atrial fibrillation (POAF).

As shown in Fig 2, AF is thought to develop via the following 2 steps: the creation of an arrhythmogenic substrate (step 1), followed by a trigger event (step 2) [11, 41]. With respect to step 1, structural remodeling (step 1A) is caused by atrial fibrosis or atrial stretch, and electrical remodeling (step 1B) is caused by the modulation of cardiac ion currents. After step 1, POAF might be triggered by multiple factors such as electrolyte disturbance, surgical inflammation, and/or cardiac autonomic imbalance (step 2). We discuss the 2 steps for POAF development below. AF is thought to develop via 2 steps: an arrhythmogenic substrate (step 1) that involves structural remodeling and/or electrical remodeling, followed by a trigger event such as electrolyte disturbance, autonomic imbalance, and/or surgical inflammation (step 2).

### Factors related to structural and electrical remodeling

In our study, factors related to structural and electrical remodeling, such as age, male sex, intraoperative fluid balance, and preoperative high-risk ECG findings, were matched even before propensity score matching. In the context of thoracic surgery, increasing age and male sex are reported preoperative risk factors for POAF [2, 4, 10]. Furthermore, increasing age is associated with atrial fibrosis [11], and the age-related prevalence of AF is less in women than in men, given that estrogen has been shown to prolong atrial action potential duration (APD) [42, 43].

Regarding perioperative management, atrial stretch due to volume overload may be associated with POAF, suggesting that intraoperative in-out fluid balance may contribute to the development of POAF [44]. In the present study, ICU specialists performed postoperative fluid management after surgery. Moreover, none of the patients developed sepsis or shock, which are also risk factors for POAF [44, 45]. The patients in the present study were comparable even before matching from the point of preoperative high-risk ECG findings, speculated to reflect atrial remodeling. Especially, increased P-wave dispersion, prolonged QRS duration, and LAE are reported to be the risk factors for POAF after lung surgery [12, 46]. For example, LAE causes interatrial electromechanical delay (electrical remodeling) related to decreased calcium flow [47].

### Factors related to the trigger of POAF

Electrolyte disturbance is a factor related to the trigger of POAF [41]. However, little research on electrolyte derangement has been performed in the context of lung surgery, in contrast to cardiac surgery [48]. Gong et al. reported an association between POAF and higher potassium concentration in lung surgery [28]. The present study matched the serum potassium level on POD1 to minimize the influence.

The autonomic nervous system plays an important role in initiating and maintaining POAF [11, 18, 49, 50]. Amar et al. [51] concluded that one of the pathogenesis of POAF in lung surgery is increased parasympathetic nervous activity with sympathetic predominance. We did not examine heart rate variability in the present study. However, pain control, which strongly correlates with sympathetic nerve activity, was performed uniformly by the acute pain service team in the propofol and desflurane groups.

Some reports demonstrated that mediastinal lymph node dissection was associated with POAF [33, 52], although there was no association found between them in this study (*p* = 0.765 in nodal dissection 0/1 vs. 2/3). In addition, surgical inflammation around the vagal nerve and pericardium with mediastinal lymph node dissection may be supposed to trigger POAF [53], but the previous studies included open approaches and pneumonectomy in contrast to our study.

### Potential mechanism of POAF inhibition by propofol: Attenuation of electrical remodeling and prevention of trigger event

Among cardiac ion currents, potassium plays an important role in human atrial myocytes by modulating repolarization (electrical remodeling). Ultrarapid-delayed-rectifier potassium current (*I*_Kur_) and transient-outward potassium current (*I*_to_) are major outward currents existing in the human atrium [54] but not the ventricle [55]. Thus, *I*_Kur_ and *I*_to_ are a target for developing anti-AF therapy [56, 57]. Commonly distributed in plants, Acacetin prolongs APD by suppressing *I*_Kur_ and *I*_to_ in human atrial myocytes and preventing AF in anesthetized dogs [58]. Similarly, propofol is demonstrated to prolong human atrial APD by inhibiting *I*_Kur_ and *I*_to_ in a dose-dependent manner [59, 60]; therefore, propofol might act as an anti-arrhythmogenic agent in the clinic, like Acacetin. In addition, the solvent for propofol contains n-3 α-linolenic acid, which blocks the human voltage-gated Kv1.5 channel, leading to *I*_Kur_ modulation [61]. Desflurane has not been reported to have modulating effects on atrial APD.

In the trigger event of AF, propofol has decreased vagal nerve activity [62] and P-wave dispersion compared to volatile anesthetic agents [36]. It suggests propofol might modulate cardiac autonomic imbalances and improve atrial electrophysiologic heterogeneity compared to volatile anesthetic agents. The present study did not evaluate the postoperative neural and electrical changes; however, ICU specialists should have performed postoperative care uniformly in the propofol and desflurane groups.

### Surgical procedure and clinical outcome

Whether the type of surgical procedure affects the incidence of POAF remains controversial [6, 7, 10, 63, 64]. In the present study, surgical procedures, such as left or right resection, did not affect POAF incidence (*p* = 0.389 in POAF vs. Non-POAF, and *p* = 0.171 in propofol vs. desflurane). Neither the magnitude of resection also affected POAF incidence (*p* = 0.763 in POAF vs. Non-POAF, and *p* = 0.769 in propofol vs. desflurane) (S2 Data). For the relation between surgical location and incidence of embolism, 2 cohort studies have identified left-upper lobectomy as a risk factor for stroke [63, 65]. None of the patients with POAF in the present study developed atherothrombotic brain infarction during their hospital stay.

From a pathological standpoint, we included patients with small cell lung cancer (n = 10), not only non-small cell lung cancer and so on. (n = 472). Although the general condition tends to be severe in small cell lung cancer by the rapid progression of its pathophysiology, they were classified as ASA-PS 1 or 2 and did not cause POAF in the current study.

### Strengths and limitations

Strengths of the present study include the fact that we evaluated a common postoperative arrhythmia after lung surgery in a homogeneous population. In addition, our results provide anesthesiologists with important information for selecting an anesthetic agent in lung surgery, considering the various perioperative complications of POAF [1–4, 27].

Whereas this study presents novel findings on the incidence of POAF between propofol and desflurane, there are a few limitations. First, anesthetic management was performed by each anesthesiologist at their discretion. Nonetheless, patients’ backgrounds were comparable between the two groups, and anesthetic management was not highly variable. Second, we might not have detected POAF after POD2 because POD2 terminated ECG monitoring. Some studies have suggested that the peak onset of postoperative dysrhythmias is a few days after surgery [1, 10]. Therefore, we focused on the effect of an anesthetic agent on immediate POAF; any potential slow-onset POAF was not assessed. Third, we included patients with and without preoperative beta-blocker medication, which was the risk factor associated with POAF by our multivariate logistic regression analysis. However, we matched the factor with the propensity score and confirmed that patients taking beta-blockers before surgery continued them in the postoperative period to avoid beta-blockade withdrawal [1], keeping hemodynamic stability [66].

## Conclusion

Our retrospective data suggest that propofol anesthesia exerts significant inhibition of POAF compared to desflurane in patients with lung disease who underwent VATS. Although we did not identify the mechanism of propofol on inhibition of POAF incidence, our results correspond with the literature. Prospective studies are needed to elucidate this effect and the mechanism of propofol inhibition on POAF.

## Supporting information

S1 File Database with raw data. (XLSX)

S1 Data Love plot for standardized mean difference before and after propensity score matching among covariances. The standardized mean difference distribution of many covariances was lower than 0.2 after propensity score matching. ASA PS: American Society of Anesthesiologists Physical Status.
(DOCX)

S2 Data Surgical site and the POAF incidence. Data are presented as number (percent). POAF: postoperative atrial fibrillation.
(DOCX)
