# Supplementary material for: Propofol anesthesia decreases the incidence of new-onset postoperative atrial fibrillation compared to desflurane in patients undergoing video-assisted thoracoscopic surgery: A retrospective single-center study
Source: PLoS One. 2023 May 2;18(5):e0285120. doi: 10.1371/journal.pone.0285120 (PMC10153745; doi:10.1371/journal.pone.0285120)
Supplement: S1 Data — The standardized mean difference distribution of many covariances was lower than 0.2 after propensity score matching. ASA PS: American Society of Anesthesiologists Physical Status. (DOCX) [file pone.0285120.s002.docx]

**Supplement 1: Love plot for standardized mean difference before and after propensity score matching among covariances.**


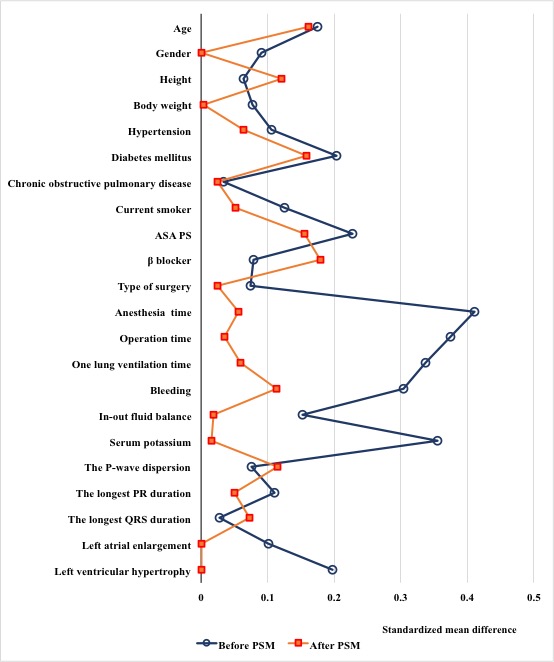


The standardized mean difference distribution of many covariances was lower than 0.2 after propensity score matching.

ASA PS: American Society of Anesthesiologists Physical Status
