# Supplementary material for: Propofol anesthesia decreases the incidence of new-onset postoperative atrial fibrillation compared to desflurane in patients undergoing video-assisted thoracoscopic surgery: A retrospective single-center study
Source: PLoS One. 2023 May 2;18(5):e0285120. doi: 10.1371/journal.pone.0285120 (PMC10153745; doi:10.1371/journal.pone.0285120)
Supplement: S2 Data — Data are presented as number (percent). POAF: postoperative atrial fibrillation. (DOCX) [file pone.0285120.s003.docx]

**Supplement 2. Surgical site and the POAF incidence.**

|  | | **POAF (n = 12)**  **N (%)** | **Non-POAF (n = 470)**  **N (%)** |
| --- | --- | --- | --- |
| **Left** | **Upper lobectomy** | 4 (33.3) | 75 (16.0) |
|  | **Lower lobectomy** | 1 (8.3) | 72 (15.3) |
|  | **Segmentectomy** | 1 (8.3) | 30 (6.4) |
| **Right** | **Upper lobectomy** | 3 (25.0) | 141 (30.0) |
|  | **Middle lobectomy** | 0 (0) | 33 (7.0) |
|  | **Upper middle lobectomy** | 0 (0) | 4 (0.9) |
|  | **Middle lower lobectomy** | 0 (0) | 3 (0.6) |
|  | **Lower lobectomy** | 3 (25.0) | 86 (18.3) |
|  | **Segmentectomy** | 0 (0) | 26 (5.5) |
| ***p* = 0.763 in POAF vs. Non-POAF** | | | |
|  | | | |
|  | | **Propofol (n=344)**  **N (%)** | **Desflurane (n=138)**  **N (%)** |
| **Left** | **Upper lobectomy** | 51 (14.8) | 28 (20.3) |
|  | **Lower lobectomy** | 54 (15.7) | 19 (13.8) |
|  | **Segmentectomy** | 19 (5.5) | 12 (8.7) |
| **Right** | **Upper lobectomy** | 104 (30.2) | 40 (29.0) |
|  | **Middle lobectomy** | 24 (7.0) | 9 (6.5) |
|  | **Upper middle lobectomy** | 3 (0.9) | 1 (0.7) |
|  | **Middle lower lobectomy** | 3 (0.9) | 0 (0) |
|  | **Lower lobectomy** | 62 (18.0) | 27 (19.6) |
|  | **Segmentectomy** | 24 (7.0) | 2 (1.4) |
| ***p* = 0.769 in propofol vs. desflurane** | | | |

Data are presented as number (percent).

POAF: postoperative atrial fibrillation
